# IL-6 signaling drives self-renewal and alternative activation of adipose tissue macrophages

**DOI:** 10.3389/fimmu.2024.1201439

**Published:** 2024-02-28

**Authors:** Jan Ackermann, Lilli Arndt, Janine Fröba, Andreas Lindhorst, Markus Glaß, Michaela Kirstein, Constance Hobusch, F. Thomas Wunderlich, Julia Braune, Martin Gericke

**Affiliations:** ^1^ Institute of Anatomy, Leipzig University, Leipzig, Germany; ^2^ Institute of Anatomy and Cell Biology, Martin-Luther-University Halle-Wittenberg, Halle (Saale), Germany; ^3^ Institute of Molecular Medicine, Martin Luther University Halle-Wittenberg, Charles Tanford Protein Center, Halle (Saale), Germany; ^4^ Max-Planck-Institute for Metabolism Research, Research Group for Obesity and Cancer, Cologne, Germany

**Keywords:** IL-6, adipose tissue inflammation, macrophages, self-renewal, obesity, diabetes, mannose receptor, alternative activation

## Abstract

**Introduction:**

Obesity is associated with chronic low-grade inflammation of adipose tissue (AT) and an increase of AT macrophages (ATMs) that is linked to the onset of type 2 diabetes. We have recently shown that neutralization of interleukin (IL)-6 in obese AT organ cultures inhibits proliferation of ATMs, which occurs preferentially in alternatively activated macrophage phenotype.

**Methods:**

In this study, we investigated AT biology and the metabolic phenotype of mice with myeloid cell-specific IL-6Rα deficiency (*Il6ra* ^Δmyel^) after normal chow and 20 weeks of high-fat diet focusing on AT inflammation, ATM polarization and proliferation. Using organotypical AT culture and bone marrow derived macrophages (BMDMs) of IL-4Rα knockout mice (*Il4ra* ^-/-^) we studied IL-6 signaling.

**Results:**

Obese *Il6ra* ^Δmyel^ mice exhibited no differences in insulin sensitivity or histological markers of AT inflammation. Notably, we found a reduction of ATMs expressing the mannose receptor 1 (CD206), as well as a decrease of the proliferation marker Ki67 in ATMs of *Il6ra* ^Δmyel^ mice. Importantly, organotypical AT culture and BMDM data of *Il4ra* ^-/-^ mice revealed that IL-6 mediates a shift towards the M2 phenotype independent from the IL-6/IL-4Rα axis.

**Discussion:**

Our results demonstrate IL-4Rα-independent anti-inflammatory effects of IL-6 on macrophages and the ability of IL-6 to maintain proliferation rates in obese AT.

## Introduction

Obesity is a worldwide growing epidemic and represents a threat to global human health by shortening life expectancy due to associated diseases as type 2 diabetes, stroke, cardiovascular disease, and cancer (1, 2). Until now, the only effective long-term treatment for obesity is bariatric surgery and there is a strong need to develop new therapeutic strategies by decoding the pathophysiology and molecular mechanisms of AT dysfunction.

In obesity, abnormal lipid accumulation leads to adipocyte hyperplasia and hypertrophy, which provokes abnormal dimensions of adipocyte death. Thereupon, the number of immune cells in AT rises, resulting in a chronic low-grade inflammation associated with type 2 diabetes and other comorbidities. Especially the number of adipose tissue macrophages (ATMs) increases as a hallmark of AT inflammation and is associated with insulin resistance (IR) (3). Additionally, the ATM immune phenotype switches from an anti-inflammatory M2-like to a pro-inflammatory M1-like state in obese mice and humans. Histologically, ATMs accumulate in so-called crown-like structures (CLS) surrounding dying adipocytes under obese conditions (4, 5). Increasing ATM number results from either CCR2-dependent recruitment of monocytes from the bloodstream or by local proliferation (6–9). Interestingly, local ATM proliferation can be stimulated by anti-inflammatory cytokines, such as IL-4 or IL-13 (9). Moreover, IL-4 treatment of obese mice affects glucose and lipid metabolism as well as insulin sensitivity in a positive manner (10). Controversially, myeloid-specific deficiency of the IL-4Rα subunit in obese mice also resulted in an improvement of metabolic parameters and less M1-like macrophages in AT and, therefore, partially protects from AT inflammation (11). The receptor-receptor- and cytokine-receptor-interactions as well as downstream signaling cascades are complex and can differ between auto- and paracrine secretion modes or between different cell types. Therefore, cytokines often have pleiotropic effects, which are poorly understood in the pathophysiology of obesity and AT inflammation. For instance, Han and colleagues showed in 2019 that pleiotropic effects of IL-6 in AT depend on the cellular source of the cytokine (12). They found that IL-6 released from adipocytes stimulates macrophage infiltration, whereas IL-6 secreted from myeloid and muscle cells decreased the infiltration process into AT (12). Moreover, cell-type specific IL-6 release results in switches between non-canonical (classical) signaling (IL-6 binds to membrane bound IL-6Rα) and canonical (trans-signaling) using a soluble receptor variant (sIL-6Rα) (12). Dimerization of the IL-6/IL-6Rα subunit with ubiquitously expressed gp130 leads to STAT3 or STAT6 phosphorylation. Furthermore, ciliary neurotrophic factor (CNTF) and IL-30 have been described as additional low-affinity ligands of the so-called IL-6R/gp130/LIFR complex in divergent pathologies (13). In obesity, circulating IL-6 is elevated in line with TNF-α serum levels (14–16). Therefore, IL-6 was seen as a main driver of AT inflammation over years. This perception changed with evidence of anti-inflammatory effects of IL-6, like improved insulin sensitivity, alternative activation of macrophages, and an augmented *Il4ra* gene expression (17). In addition, neutralization of IL-6 in murine obese AT explants showed an inhibition of IL-4 mediated ATM proliferation combined with a decrease in *Il4ra* (9).

To further address the role of IL-6Rα signaling on ATM proliferation in obese individuals *in vivo*, we analyzed lean and obese mice with a LysM-Cre driven myeloid cell-specific deficiency of the IL-6Rα (*Il6ra* ^Δmyel^) for markers of proliferation as well as ATM polarization, general markers of AT inflammation and insulin sensitivity. Interestingly, our investigation revealed a lower percentage of CD206+ macrophages within AT and a decrease of ATM proliferation in obese *Il6ra* ^Δmyel^ mice compared to control mice. Moreover, IL-6 stimulation of organotypical AT explants and bone marrow derived macrophages (BMDMs) lacking the IL-4Rα subunit indicates that IL-6 shifts towards an M2 phenotype independent of IL-4Rα.

## Results

### Myeloid-specific *Il6ra* knockout does not affect weight gain or metabolism during protracted diet-induced obesity

**Figure 1:**
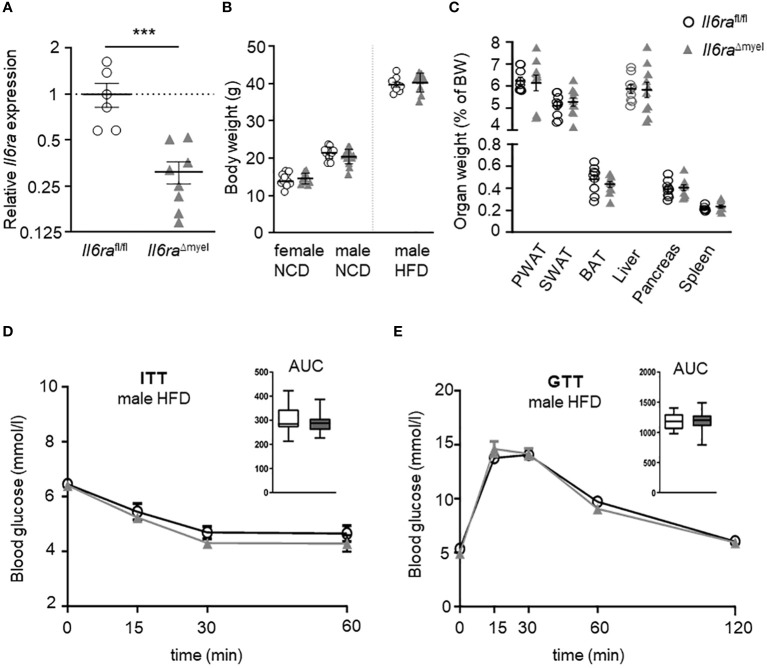
Myeloid *Il6ra* knockout does not affect weight gain and disturbed metabolism of protracted diet-induced obesity. **(A)** Relative gene expression analysis of *Il6ra* ^fl/fl^ and *Il6ra* ^Δmyel^ BMDMs to verify the *Il6ra* knockout (n=7-8). **(B)** Comparison of body weight in either lean female, lean male and obese male *Il6ra* ^fl/fl^ and *Il6ra* ^Δmyel^ mice. **(C)** Organ weights of different fat depots, liver and pancreas in obese male *Il6ra* ^fl/fl^ and *Il6ra* ^Δmyel^ after 20 weeks of HFD (n=9-16). Blood glucose measurements by ipITT **(D)** and ipGTT **(E)** of *Il6ra* ^fl/fl^ and *Il6ra* ^Δmyel^ male mice after 20 weeks of HFD (n=17). Data are presented as mean ± SEM. ***p < 0.001.

Previous studies showed that IL-6 signaling affects glucose homeostasis in a positive manner and leads to alternative activation of BMDMs (17). Furthermore, the neutralization of IL-6 in obese murine AT explant cultures leads to the inhibition of ATM proliferation (9). During obesity, serum levels of IL-6 as well as *Il6* gene expression are augmented in AT (9, 18). To verify the effect of IL-6 signaling on obesity-driven ATM proliferation *in vivo*, we used a myeloid-specific knockout of the IL-6Rα (*Il6ra* ^Δmyel^). First, IL-6Rα knockout was confirmed by a significant reduction of *Il6ra* gene expression in BMDMs measured by qRT-PCR (**Figure 1A**). Moreover, we found no differences in body weight of mice lacking the IL-6Rα compared to wildtype controls in all groups (female NCD, male NCD and male HFD) (**Figure 1B**). This might be due to utilization of different regimen for diet-induced obesity as published previously. Interestingly, lean male *Il6ra* ^Δmyel^ mice exhibited more perigonadal (PWAT) and subcutaneous (SWAT) AT compared to control mice on a chow diet, while showing no differences in food intake (**Supplementary Figures 1A-C**). However, HFD-provoked body weight gain was still present in *Il6ra* ^Δmyel^ mice (**Figure 1B**). Weight of several organs related to obesity-caused pathologies, e.g. fat depots, liver, and pancreas showed no significant differences in *Il6ra* ^Δmyel^ mice after 20 weeks of HFD (**Figure 1C**). Additionally, ipITT and ipGTT data of obese *Il6ra* ^Δmyel^ and *Il6ra* ^fl/fl^ mice revealed no differences in insulin sensitivity or glucose tolerance after HFD (**Figures 1D, E**) as well as for male mice fed a NCD (**Supplementary Figures 1D-G**; **Supplementary Table 1**). As shown in previous studies, diet-induced obesity leads to changes in gene expression in visceral AT (9). Here, we found no differences in gene expression between obese *Il6ra* ^Δmyel^ and control mice using qRT-PCR (**Supplementary Figure 2A**).

### Adipocyte expansion and ATM distribution are unaltered in obese mice lacking the IL-6Rα

**Figure 2:**
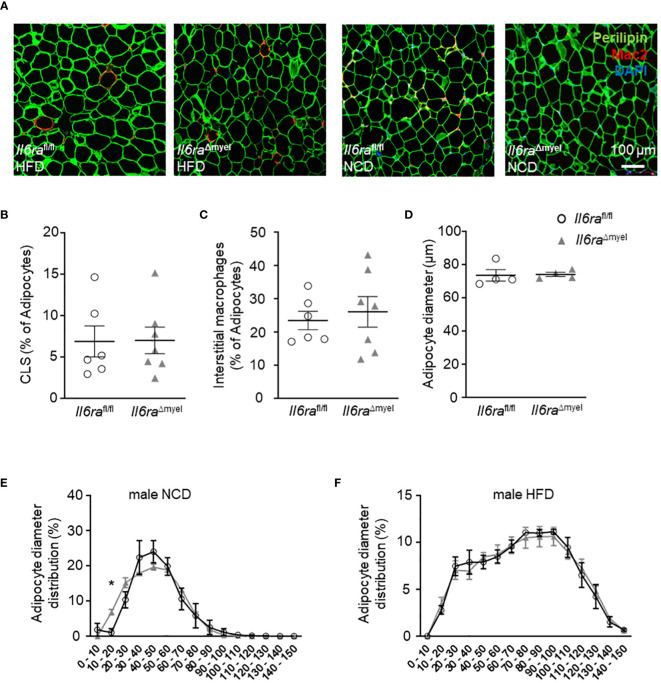
Adipocyte expansion and ATM distribution are unaltered in obese mice lacking IL-6Rα. **(A)** Representative images of immunohistochemistry of paraffin-embedded AT from obese (left side) and lean (right side) *Il6ra* ^fl/fl^ and *Il6ra* ^Δmyel^ mice stained with Perilipin (adipocytes, green), pan macrophage marker Mac-2 (red) and DAPI (nuclei, blue). **(B)** Abundance of CLS in % of adipocyte number, **(C)** interstitial macrophages in % of adipocyte number and **(D)** measurements of adipocyte size in µm from paraffin-embedded AT sections of obese *Il6ra* ^Δmyel^ and *Il6ra* ^fl/fl^ controls (n=6-7). **(E, F)** Distribution of adipocytes concerning adipocyte size in **(E)** lean and **(F)** obese male *Il6ra* ^Δmyel^ and *Il6ra* ^fl/fl^ mice. Data are presented as mean ± SEM. *p < 0.05. Scale bar represents 100 µm.

Next, we analyzed paraffin-embedded sections of lean and HFD-fed *Il6ra* ^Δmyel^ and *Il6ra* ^fl/fl^ mice stained for Perilipin to visualize adipocytes and the macrophage marker Mac-2 (**Figure 2A**). We studied macrophage distribution, formation of crown-like structures (CLS) and adipocyte size, as hallmarks of obesity induced AT dysfunction. However, analyses revealed no differences for CLS density (**Figure 2B**), percentage of interstitial macrophages per adipocyte (**Figure 2C**), or mean adipocyte diameter (**Figures 2D-F**) in lean and obese *Il6ra* ^Δmyel^ compared to *Il6ra* ^fl/fl^ mice. Flow cytometry analysis verified normal leukocyte and ATM enhancement after HFD in both mouse lines (**Supplementary Figures 2B, C**). Diet-induced obesity is concomitant with altered levels of triglycerides, free fatty acids, or cholesterol. In this study, we found no significant changes in these parameters comparing HFD *Il6ra* ^Δmyel^ and their littermate controls (**Supplementary Table 1**).

### Myeloid *Il6ra* knockout impairs alternative activation and proliferation of ATMs in diet-induced obesity

**Figure 3:**
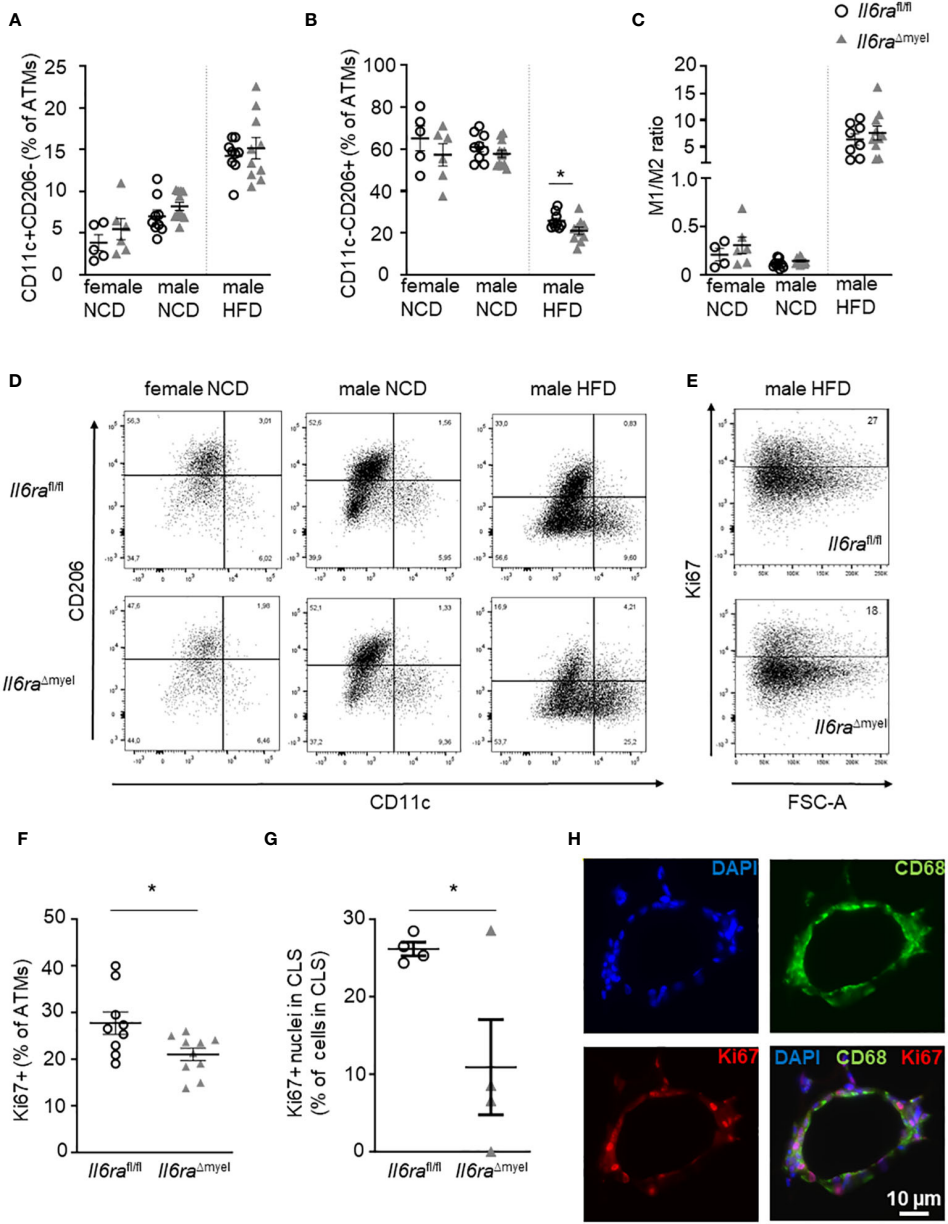
Myeloid *Il6ra* knockout impairs alternative activation and proliferation of ATMs in diet-induced obesity. **(A, B)** Flow cytometry analysis of classically activated M1 (A; CD11c+CD206-) and alternatively activated M2 (B, CD11c-CD206+) given as percentage of overall ATMs (CD45+F4/80+) in AT of lean and obese female and obese male *Il6ra* ^fl/fl^ and *Il6ra* ^Δmyel^ mice (either NCD or 20 weeks of HFD; n=6-12). **(C)** Ratio of M1 to M2 macrophages in female and male NCD and HFD *Il6ra* ^fl/fl^ and *Il6ra* ^Δmyel^ mice measured by flow cytometry (n=4-12). **(D)** Representative flow cytometry plots of female and male *Il6ra* ^fl/fl^ and *Il6ra* ^Δmyel^ mice under NCD or HFD reflecting CD11c and CD206 expression in ATMs. **(E)** Representative flow cytometry plots for Ki67 expression on overall ATMs (CD45+F4/80+) in obese AT of *Il6ra* ^fl/fl^ and *Il6ra* ^Δmyel^ mice. **(F)** Percentage of Ki67+ ATMs (CD45+F4/80+) in AT of obese *Il6ra* ^fl/fl^ and *Il6ra* ^Δmyel^ mice (n=6-11). **(G)** Ki67+ nuclei within CLS as percentage of all nuclei in CLS using paraffin-embedded AT sections of obese male *Il6ra* ^fl/fl^ and *Il6ra* ^Δmyel^ mice (n=4). **(H)** Representative images of an obese CLS with staining of macrophages (CD68, green), nuclei (blue) and Ki67 (red). Scale bar represents 10 µm. Data are presented as mean ± SEM. *p < 0.05.

Although we could not detect any differences in CLS formation, ATM distribution, and adipocyte size (**Figures 2B-F**), we hypothesized that IL-6 signaling in myeloid cells impacts on the activation state of ATMs and their proliferation as described before *ex vivo* (9). To test this hypothesis, we analyzed ATMs (CD45+F4/80+) using flow cytometry (see gating strategy **Supplementary Figure 4**). Obesity is associated with an augmentation in AT leukocytes, especially ATMs. We found diet-induced enhancement of these cell types independent from IL-6Rα depletion (**Supplementary Figures 2B, C**; **Supplementary Table 1**). Next, we tested the ATM expression of the pro-inflammatory marker CD11c (Integrin alpha-X; encoded by the *Itgax* gene) and the anti-inflammatory marker CD206 (Mannose receptor 1; encoded by the *Mrc1* gene) to detect alterations in the activation state of ATMs between *Il6ra* knockout and wildtype mice (**Figures 3A-D**). Here, we defined CD11c+CD206- as pro-inflammatory M1 macrophages, whereas CD11c-CD206+ ATMs were defined as M2-like macrophages. Interestingly, we could not detect an impact of disrupted IL-6 signaling on CD11c+CD206- expressing ATMs, neither in NCD nor HFD mice (**Figures 3A, D**; **Supplementary Table 1**). Hence, the increase of CD11c+CD206- macrophages in AT due to obesity-related inflammation is still existent in mice lacking the IL-6Rα in myeloid cells (**Figures 3A, D**). Importantly, CD206 expression in ATMs of obese *Il6ra* ^Δmyel^ mice is reduced, whereas IL-6Rα knockout has no influence in lean individuals (**Figures 3B, D**). Of note, the impact of IL-6 signaling on CD206 expression is also reflected by increased CD11c-CD206- and decreased CD11c+CD206+ ATM populations in obese *Il6ra* ^Δmyel^ mice (**Supplementary Figures 2D, E**). The overall activation state of ATMs reflected by the M1/M2 ratio is not significantly affected by disrupted IL-6 signaling (**Figures 3C, D**).

*Ex vivo* data suggest a role of IL-6 signaling in ATM proliferation. Hence, we measured the percentage of all ATMs expressing the proliferation marker Ki67 within obese AT comparing *Il6ra* ^Δmyel^ and their control littermates by flow cytometry (**Figures 3E, F**) or by microscopy (**Figures 3G, H**). In accordance with previous *ex vivo* data, proliferation of ATMs measured by Ki67 expression is significantly decreased in mice lacking IL-6Rα in myeloid cells during HFD as measured by two independent methods (**Figures 3E-H**). In lean mice, we found no difference in ATM Ki67 expression (**Supplementary Table 1**). Of note, with our protocol we were not able to detect changes in Bromodesoxyuridine (BrdU) incorporation in AT leukocytes of obese *Il6ra* ^Δmyel^ and control animals (**Supplementary Figures 2F, G**). However, also different T-cell populations can affect ATM polarization and proliferation, which in turn affects insulin sensitivity and the outcome of diet-induced obesity (19–21). Therefore, we analyzed T-cell subsets isolated from obese AT of either *Il6ra* ^Δmyel^ or *Il6ra* ^fl/fl^ mice fed a NCD or HFD (**Supplementary Figure 3**). However, by comparing male *Il6ra* ^Δmyel^ and *Il6ra* ^fl/fl^ mice on a HFD, we could not detect significant differences (**Supplementary Figures 3A, D**; **Supplementary Table 1**). We also measured the expression of ST2 and FoxP3 in CD4+ T-cell populations to quantify prevalence of Th2 and regulatory T-cells. Here, we also did not find differences in male HFD mice with or without an intact IL-6Rα subunit (**Supplementary Table 1**).

### IL-6 elevates proliferation of anti-inflammatory M2 macrophages partially independent of the IL-4Rα

**Figure 4:**
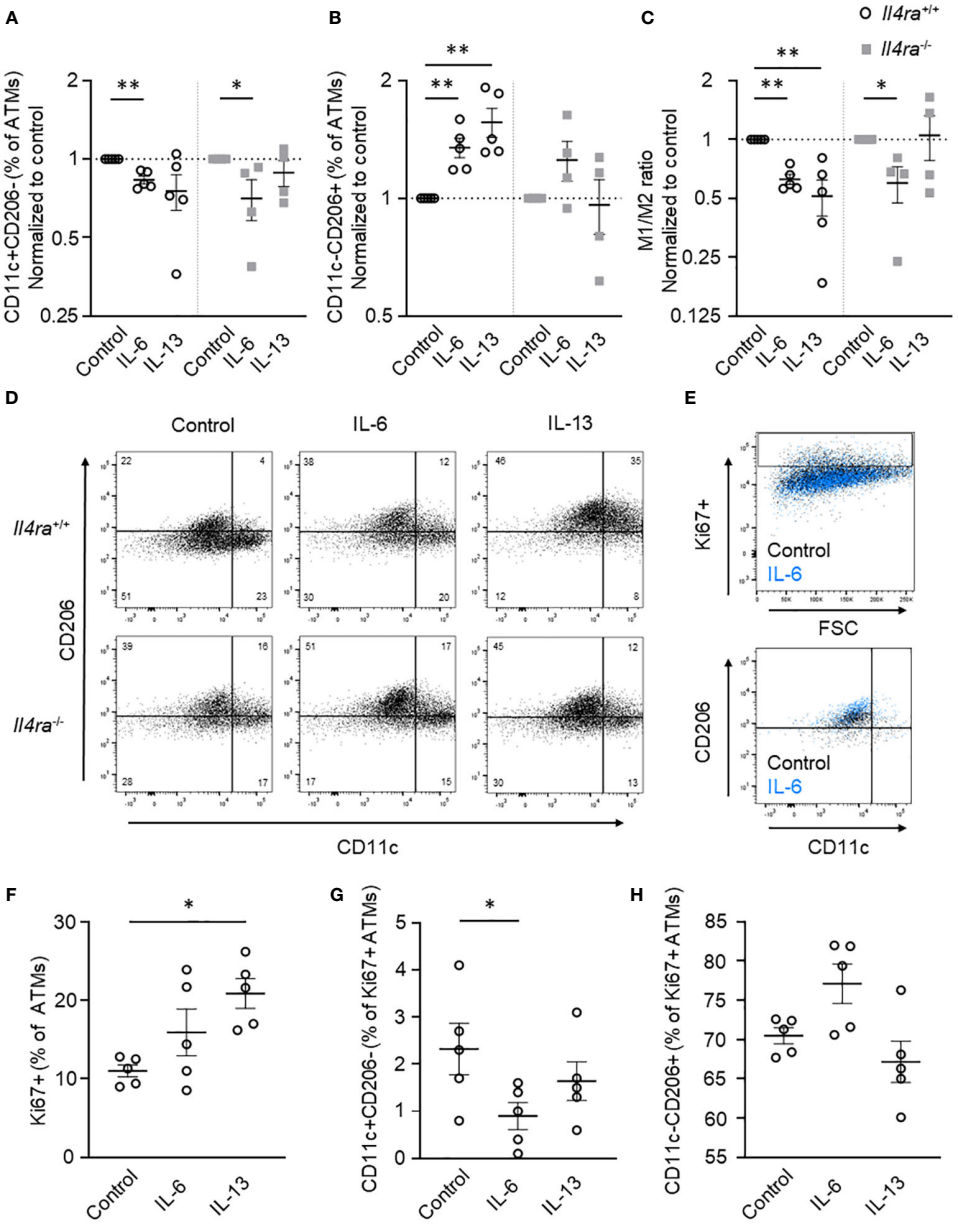
IL-6 elevates anti-inflammatory M2 macrophages partially independent of the IL-4Rα. **(A-D)** Flow cytometry data of AT explants from lean male *Il4ra* ^+/+^ (wildtype) and *Il4r* ^-/-^ (knockout) mice after *ex vivo* induction of AT inflammation showing **(A)** CD11c+CD206- (M1) macrophages, **(B)** CD11c-CD206+ (M2) macrophages and **(C)** M1/M2 ratio after treatment with IL-6 or IL-13 (50 ng/ml) for 48h (n=4-5). **(D)** Representative flow cytometry plots for IL-6 and IL-13 treatment in lean inflammatory AT of male and female *Il4ra* ^+/+^ and *Il4r* ^-/-^ mice. **(E-H)** Proliferation measurements using Ki67 staining and flow cytometry in lean male and female *Il4ra* ^+/+^ inflammatory AT after IL-6 and IL-13 stimulation (50 ng/ml, 48h; n=4-5). **(E)** Representative flow cytometry plots of IL-6 stimulated AT explants after induction of inflammation (control black, IL-6 blue events). **(F)** Ki67+ cells as percentage of overall ATMs. **(G)** CD11c+CD206- und **(H)** CD11c-CD206+ proliferating ATMs as percentage of Ki67+ ATM. Data are presented as mean ± SEM. *p < 0.05, **p < 0.01.

In previous studies, alternative activation of ATMs as well as ATM proliferation was linked to IL-6 signaling *via* the IL-4Rα-Stat6-axis (9, 17). To verify IL-6-mediated impact on ATM polarization in inflammatory AT and to get more insights into the interrelation with IL-4Rα signaling, we investigated the effect of IL-6 stimulation in AT of Il4ra^+/+^ (wildtype) and Il4ra^-/-^ (knockout) mice. To mimic obesity-induced AT inflammation in lean individuals, AT explants were cultured for 7d to induce adipocyte death, CLS formation and augmentation of the pro-inflammatory M1 phenotype as shown before (9). Treatment of AT organ culture with IL-6 (50 ng/ml) for 48h resulted in a significant decrease of CD11c+CD206- ATMs and a significant increase of CD11c-CD206+ ATMs as measured by flow cytometry (**Figures 4A-D**) confirming our results from myeloid-specific IL-6Rα examinations. Of note, our *Il4ra* knockout model showed a variation in the baseline of ATM polarization due to global IL-4Rα deficiency. Therefore, data were normalized to the respective PBS control. Stimulation of AT explants with IL-13, a potent ligand of IL-4Rα, generated conventionally M2-polarized ATMs for comparison in control AT and, on the other hand, confirmed efficient knockout in IL-4Rα-deficient AT (**Figures 4A-D**). Most importantly, upon IL-6 stimulation the ratio of M1 to M2 ATMs shifted towards M2 irrespective of IL-4Rα deficiency, indicating that IL-6 can stimulate M2 polarization without involvement of the IL-4Rα axis (**Figures 4A-D**). Moreover, we investigated the influence of IL-6 stimulation on ATM proliferation. In our explant model, we could only detect a trend towards higher Ki67 expressing ATMs after IL-6 stimulation, which can probably be explained by intrinsically high IL-6 levels (**Figures 4E, F**) as discussed elsewhere (9). Of note, IL-6 treatment shows significant reductions in proliferative M1 macrophages (**Figures 4G**) and a trend towards a more proliferative M2 ATM phenotype, indicating preferential M2 polarization of proliferating ATMs (**Figures 4G, H**).

### IL-6 signaling boosts CD206 expression partially independent of the IL-4Rα subunit

**Figure 5:**
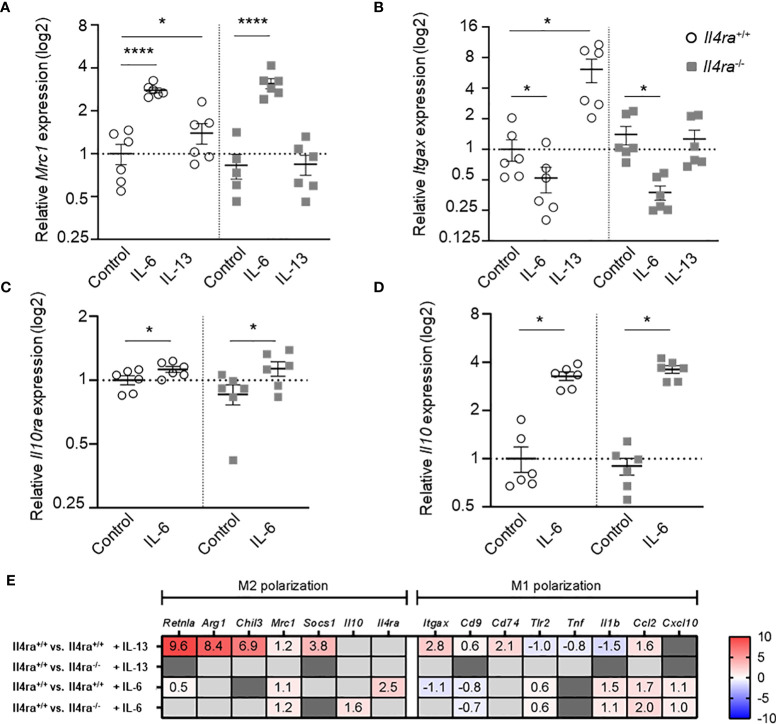
IL-6 signaling boosts BMDM *Mrc1* expression and decreases *Itgax* expression independently of the IL-4Rα subunit. **(A, B)** Relative gene expression data of *Mrc1* (A, Mannose receptor 1) and *Itgax* (B, Integrin alpha X) in BMDMs (M0) from non-obese male *Il4ra* ^-/-^ or control (*Il4ra* ^+/+^) mice after stimulation with 20 ng/ml IL-13 or IL-6 for 48h (n=5-6). **(C, D)** Relative gene expression of *Il10ra* and *Il10* of IL-6 treated (20 ng/ml) BMDMs from non-obese male *Il4ra* ^-/-^ or wildtype (*Il4ra* ^+/+^) mice. PBS served as control. Gene expression data were related to *Importin-8* (*Ipo8*) as internal control (n=6). **(E)** Heat map of RNA sequencing data depicting only significantly regulated genes of *Il4ra* ^-/-^ or control (*Il4ra* ^+/+^) BMDMs treated with PBS, IL-13 or IL-6 (20 ng/ml) for 48h as log2FC (n=4). Genes of interest were assigned to alternatively activated (M2) and classically activated (M1) genes. Data are presented as mean ± SEM. *p < 0.05, ****p < 0.0001.

To get further insights into the IL-6-induced macrophage phenotype and to study the role of the IL-4Rα in IL-6 signaling in more detail, we performed gene expression analysis and bulk RNA sequencing of *Il4ra* ^+/+^ and *Il4ra* ^-/-^ BMDMs stimulated with IL-13 and IL-6 (**Figures 5A, B**). Alternative activation and, therefore, an anti-inflammatory M2 phenotype of macrophages is closely linked to the expression of macrophage mannose receptor 1 (CD206) encoded by the *Mrc1* gene. In contrast, the pro-inflammatory M1 phenotype is associated to CD11c expression on macrophages, which is encoded by the *Itgax* gene. By IL-13 stimulation of BMDMs from *Il4ra^+/+^* and *Il4ra* ^-/-^ mice, we were able to generate conventionally M2-polarized BMDMs and to verify the *Il4ra* knockout by abrogated IL-13 signaling (**Figures 5A, B**). Interestingly, after stimulation with IL-6 a similar enhancement of *Mrc1* gene expression was seen in both, control as well as *Il4ra* knockout mice (**Figure 5A**). qRT-PCR also revealed a decrease of *Itgax* expression in control and *Il4ra* ^-/-^ BMDMs after IL-6 stimulation (**Figure 5B**). IL-6 is known to increase IL-10 production and alternative activation of macrophages was described to be closely linked to signaling *via* IL-10 and IL-10Rα (22). Therefore, we tested whether the IL-6-dependent increase of *Il10* relies on IL-4Rα signaling by stimulation of *Il4ra* ^-/-^ BMDMs with IL-6, which revealed a similar increase in *Il4ra* ^-/-^ BMDMs (**Figures 5C, D**).

To gain further insights into macrophage gene expression patterns after IL-13 or IL-6 stimulation and the dependence on IL-4Rα signaling, we performed bulk RNA sequencing analysis of BMDMs derived from *Il4ra* ^-/-^ and control mice (the 100 most differentially expressed genes are shown in **Supplementary Figure 5**). In **Figure 5E**, log_2_ fold changes of significantly regulated genes of interest are shown and allocated to the M2 or M1 phenotype. IL-13 stimulation of BMDMs, derived from wildtype mice, induces M2-like as well as M1-like gene expression (**Figure 5E**, RNA sequencing data accession PRJNA971096), as shown in our previous study (11). Only very few IL-13-mediated effects are detectable in *Il4ra* ^-/-^ BMDMs and may be explained by other receptors (e.g. IL-13Rα2; **Supplementary Figure 5**). In line with our previous experiments, IL-6 treatment of BMDMs causes enhanced expression of *Mrc1* in both wildtype and *Il4ra* knockout mice to a similar extent (**Figure 5E**, RNA sequencing data accession PRJNA971096). In contrast to OC-AT and qRT-PCR findings, we cannot confirm the decrease of *Itgax* expression after IL-6 stimulation of *Il4ra* ^-/-^ BMDMs, which may be due to higher discovery power of isotype forms using RNA sequencing. However, the increase of *Il10* expression by IL-6 was even more pronounced in *Il4ra* ^-/-^ BMDMs (**Figure 5E**).

Moreover, differential gene expression (DEG), gene set enrichment analysis (GSEA) and gene ontology (GO) analysis underlined interruption of IL-13 signaling in *Il4ra* ^-/-^ BMDMs compared to wildtype control (**Supplementary Table 3**, **4**). In contrast, IL-6 stimulation of *Il4ra* ^-/-^ BMDMs shows comparable results compared to control BMDMs further underlining IL-4Rα-independent anti-inflammatory signals of IL-6 (**Supplementary Table 3**, **4**).

## Discussion

AT of obese individuals exhibits a chronic low-inflammatory state, linked to the onset of type 2 diabetes and other comorbidities. This chronic low-grade inflammation is characterized by an altered composition of ATM phenotypes, an augmented ATM number, and local proliferation of ATMs (9, 23). Interestingly, local proliferation of macrophages is preferentially found in anti-inflammatory macrophages and seems necessary for maintaining tissue homeostasis and refreshing the tissue microenvironment (23). Of note, local proliferation of ATMs can be linked to IL-6 signaling as shown before in *ex vivo* AT organ culture studies (9). In subjects with overweight and obesity, circulating levels of IL-6 are found to be augmented (24). Therefore, IL-6 was thought to be a driver of AT inflammation and associated with the activation of ATMs towards the pro-inflammatory M1 phenotype. Most importantly, Mauer and colleagues found evidence for an anti-inflammatory role of IL-6 in AT of obese mice. They reported a decreased number of alternatively activated macrophages in obese AT and a deterioration of insulin sensitivity in mice lacking the IL-6Rα (17). These findings are in line with recent studies suggesting anti-inflammatory effects of IL-6 in other diseases, like cancer and neuro-inflammation (25, 26). Here, our group investigated the correlation of ATM proliferation and polarization with IL-6 signaling *in vivo* using myeloid cell-specific IL-6Rα-deficient adult mice after 20 weeks of HFD.

We found no significant differences in hallmarks of obesity related AT inflammation analyzing *Il6ra* ^Δmyel^ and *Il6ra* ^fl/fl^ mice using AT histology to examine CLS formation, macrophage distribution or numbers as well as adipocyte size, which is in contrast to previous studies (17). This may be explained by different study protocols, e.g. divergent ages of mice (23 weeks vs. 28 weeks), HFD protocols (starting week 3 of age vs. starting at week 8 of age), the circadian rhythm of mice or different animal facilities, which impacts on microbiota (27–31).

Analyzing ATM subsets from AT of obese *Il6ra* ^Δmyel^ and *Il6ra* ^fl/fl^ mice reveals a reduced number of CD206-positive ATMs in knockout mice, which matches our previous reports approving beneficial effects of IL-6 signaling on alternative ATM activation (9). Additionally, the subset of CD11c+CD206+ ATMs, which is a marker of ongoing AT inflammation and correlates to insulin resistance, was decreased in AT of *Il6ra* ^Δmyel^ mice (32).

Noteworthy, the M1/M2 classification is an oversimplification based on cell culture work and ATMs show quite diverse phenotypes and physiological characteristics (33). Beside the M1/M2 paradigm, ATMs are also grouped to metabolically active ATMs, CD9+ ATMs, TREM2+ lipid associated macrophages (LAM) and sympathetic neuron-associated macrophages (SAM) (34–37). Whether occurrence and/or function of either of these ATM subsets is affected by IL-6 signaling needs to be studied in more detail in the future, e.g. by using single cell RNA sequencing.

Stimulation of AT explants with IL-13, a typical Th2 cytokine, leads to an augmentation of macrophages with CD11c and CD206 expression as shown before (11). In contrast, IL-6 treatment of BMDMs shows anti-inflammatory effects by enhancing *Mrc1* and *Il10* expression and lowering *Itgax* gene expression. In this study, we detected lower levels of Ki67 expression in obese *Il6ra* ^Δmyel^ mice, which confirms pro-proliferative effects of IL-6Rα signaling on macrophages. Additionally, IL-6 treatment of AT explants with *ex vivo* generated AT inflammation shows a trend to more proliferative macrophages, which seems to boost anti-inflammatory M2 macrophage proliferation and significantly lowers M1 renewal. Notably, we were unable to support this result by our BrdU incorporation protocol, maybe due to limitation of BrdU incorporation to active DNA replication. Of note, BrdU incorporation preferentially labels proliferating cells in S phase of the cell cycle (38), whereas Ki67 expression depicts cells also during G2 and M phase (39).

In our previous study, we speculated that anti-inflammatory polarization and proliferation of ATMs stimulated by IL-6 is dependent on the IL-4Rα (9), since the activation of the IL-4Rα subunit by IL-4 and IL-13 is the most common pathway for alternative macrophage activation (40–42). Therefore, we tested IL-6 signaling in ATMs of an AT inflammation tissue inflammation model as well as in BMDMs of mice with a global knockout of this IL-4/IL-13 receptor subunit. Surprisingly, IL-6 stimulation lowers the percentage of the M1 subpopulation in ATMs and increases the number of M2 macrophages due to stimulation of M2 proliferation. These effects can also be observed in mice lacking the IL-4Rα, whereat it should be mentioned that IL-6-driven macrophage polarization strongly depends on the polarization state prior to IL-6 stimulation (43). Additionally, our *ex vivo* AT inflammation model cannot fully imitate obesity-associated AT inflammation e.g. due to missing recruitment of leukocytes from the bloodstream. But also IL-6 treatment of BMDMs leads to the enhancement of *Mrc1* and *Il10* expression independently of the IL-4Rα. Taken together this study describes a new mechanism for alternative activation of macrophages by IL-6 independently of the IL-4Rα-Stat6-axis.

RNA sequencing data revealed IL-4Rα-independent changes in RNA levels of pro- and anti-inflammatory macrophage genes after IL-6 stimulation of wildtype versus *Il4ra* ^-/-^ mice. Consistent with the finding of Mauer et al., IL-6 stimulation of BMDMs induces the expression of the *Il4ra* which was confirmed by qRT-PCR (17). Of note, IL-6-mediated effects on alternative activation of macrophages can be enhanced by treating BMDMs that were pre-stimulated with IL-4 and IL-13 (17, 43). In our model of diet-induced obesity, a substantial proportion of ATMs is M1 polarized. However, since effects of IL-6 signaling might depend on M2 ATM occurrence, pathophysiological effects of IL-6Rα depletion, such as changes in glucose tolerance, might depend on the duration of pre-existing AT inflammation. Hence, a shorter HFD, as Mauer and colleagues utilized in their study, could imply a greater M2 population as mediator of disrupted IL-6 signaling. Further, we are also unable to exclude pro-inflammatory effects of a soluble mannose receptor variant (25). In addition, the source of IL-6 secretion seems to play an important role for its action, whereby adipocyte-derived IL-6 triggers macrophage recruitment. In contrast, IL-6 secretion by myeloid cells suppresses macrophage recruitment from the blood stream in obese AT (12). Thus, a mouse model with a conditional cell-specific overactivation of IL-6 signaling in macrophages could be a promising model to further investigate the role of IL-6 in obesity. Nevertheless, in this study myeloid cell-specific deficiency of IL-6Rα in obese mice leads to a decline in ATM proliferation and CD206 protein expression in ATMs indicating anti-inflammatory effects of IL-6 signaling on ATMs. The participation of other myeloid cells, such as neutrophils, in long-term HFD-induced AT inflammation seems unlikely due to low quantity (~1% of stroma cells) but cannot be excluded due to LysM-driven recombination in neutrophils (26). Since CD206 is also described to mediate phagocytosis (44, 45), augmentation of CD206 on ATMs could be helpful in the resolution of dying adipocytes and AT inflammation. Owing to IL-6-mediated effects on ATM proliferation, polarization and recruitment, IL-6 and mannose receptor 1 expression sustain potential targets to rescue insulin sensitivity in obesity and the role of IL-6 as a pro-inflammatory clinical parameter should be revised.

## Materials and methods

### Experimental animals

Mice strains were maintained in pathogen-free facilities at the University of Leipzig on a 12-h light/dark cycle at 22 ± 2°C with free access to food and water. For diet-induced obesity, *Il6ra* ^fl/fl^ x LysM-Cre^-/+^ (*Il6ra* ^Δmyel^) or wildtype (*Il6ra* ^fl/fl^) littermate controls on a 57BL/6N background were fed a high-fat diet (HFD) (60% kcal fat; Ssniff Spezialdiäten) for 20 weeks starting at the age of 8 weeks. Control littermates as well as *Il4ra* ^+/+^ and *Il4ra* ^-/-^ mice were kept on a regular chow diet (9% kcal fat; Ssniff Spezialdiäten; Soest, Germany).

Perigonadal (PWAT), subcutaneous (SWAT) and brown adipose tissue (BAT), liver, pancreas, spleen, and brain were dissected, weighed and either shock frozen in liquid nitrogen or fixed in zinc formaldehyde (Polyscience; Warrington, PA, USA; 21516-3.75) for further examination. All experiments were approved by the local authorities of the state of Saxony (TVV 11/18; Landesdirektion Leipzig, Germany).

### Induction of *ex vivo* AT inflammation

For mimicking AT inflammation as seen in obesity, organotypic organ-cultures of lean visceral AT were generated as described before (9). Explants were placed under a sterile cell culture insert (pore size 0.4 µm; Sarstedt, Nümbrecht, Germany) and cultured in RPMI 1640 medium (Sigma-Aldrich, Deisenhofen, Germany) supplemented with 1% insulin/transferrin/selenium mixture, antibiotics (100 U/ml penicillin and streptomycin; all reagents from Sigma-Aldrich) and 10% fetal bovine serum at 5% CO_2_, 21% O_2_ and 37°C for 7 days without any intervention. Subsequently, AT explants were treated with PBS, IL-6 or IL-13 (50 ng/ml) for 48h.

### Immunofluorescence and H&E staining

After fixation, AT was embedded in paraffin as described previously (22, 29). At 4°C sections were incubated overnight with primary antibodies anti-Mac-2 (1:1000; Cedarlane; Burlington, ON, Canada; CL8942AP) and anti-PerilipinA (1:200; Abcam; Cambridge, UK; ab3526). Next, fluorochrome-conjugated secondary antibodies were applied for 1 h at room temperature (1:200; Invitrogen; Waltham, MA, USA). For nuclear staining DAPI (1:10,000; Thermo Fischer Scientific; Waltham, MA, USA; 62248) was used. For control stainings, same routines without primary antibodies were applied. A confocal Leica SPE microscope (Leica; Wetzlar, Germany) was used for image acquisition. Quantification of adipocyte size, interstitial macrophages and CLS density were performed semi-automatically with cellSens Software (Olympus; Hamburg, Germany) as described previously (30). H&E stainings were performed following standard routines (29).

### Metabolic characterization

Mice were weighed weekly, starting at 5 weeks of age until euthanization. At ages of 8 weeks (before starting HFD feeding) and 28 weeks (after 20 weeks of HFD) intraperitoneal insulin (ipITT) and glucose tolerance tests (ipGTT) were performed. For ipITT of lean mice, baseline glucose levels were measured before mice are injected intraperitoneally with insulin (Insuman Rapid, 100 IU/ml) in a concentration of 0,75 U per kg bodyweight. Blood glucose was measured again at 15, 30 and 60 min after injection. ipGTT of lean mice was performed 3 days after ipITT. Again baseline glucose levels were analyzed before mice were injected intraperitoneally with 2 g glucose per kg bodyweight (20%; B. Braun, Melsungen, Germany). Glucose levels were measured again at 15, 30, 60 and 120 min after injection. After HFD mice received for ipITT 1,5 U insulin per kg bodyweight because of a lower response to insulin. For ipGTT 1 g glucose per kg bodyweight was administered to avoid the possibility of prolonged hyperglycemia. Food intake was measured for 5 days in lean male and female *Il6ra^fl/fl^* and *Il6ra^Δmyel^* mice.

### Analytical procedures

Free fatty acids (FFA), triglycerides, total cholesterol, low-density lipoprotein- (LDL) and high-density lipoprotein- (HDL) cholesterol in plasma were determined by an automatic chemical analyzer at the Institute of Laboratory Medicine and Clinical Chemistry at the University of Leipzig.

### Culture of bone marrow-derived macrophages

To generate bone marrow-derived macrophages (BMDMs) bone marrow stems cells were flushed out of femur and tibia from respective mice. Cells were plated on 15 cm Petri dishes in RPMI-1640 medium (Sigma-Aldrich; St. Louis, MO, USA; R8758) (supplemented with 10% FCS, 1% glutamine, 1% penicillin-streptomycin) and differentiated with 20 ng/ml macrophage colony stimulating factor (M-CSF; PeproTech; Rocky Hill, NJ, USA; 315-02) for 7-10 d. Subsequently, BMDMs were stimulated with 20 ng/ml recombinant IL-6 (PeproTech; 216-16) or recombinant IL-13 (PeproTech; 210-13) for 48 h.

### Flow cytometry analysis

Immediately dissected PWAT was digested using collagenase type II (Worthington Biochemical; Lakewood, NJ, USA; LS0041-76) and filtered through a 70 µm mesh. Fc receptors were blocked for 10 min with anti-CD16/32 (1:100; eBioscience; Waltham, MA, USA; 14-0161-82). Cell stainings were performed with anti–CD45-FITC (1:200; eBioscience; 11-0451-85), anti–F4/80-PE-Cy7 (1:100; eBioscience; 25-4801-82), anti–CD11c-PE (1:100; eBioscience; 12-0114-83), anti–CD206-Alexa Fluor 647 (1:50; AbD Serotec, Kidlington, UK; MCA 2235A647), anti-CD4-PE (1:100; Biolegend; 100512), anti-CD8b-Alexa Fluor 647 (1:100; Biolegend; 126612) and/or anti-ST2-PE (1:100; eBioscience; 12-9335-82) for 20 min on ice. Cell cycle state and cell doublets were identified by staining with 7-aminoactinomycin D (7-AAD; 1:25; BD Biosciences; 552598).

Intracellular protein staining and cell proliferation assays required cell fixation and permeabilization according to the Bromodeoxyuridine (BrdU) flow kit manufacturer’s protocol (BD Biosciences; Franklin Lakes, NJ, USA; 552598), before incubation with anti-Ki67 primary antibody (SP6; 1:100; DCS Immunoline; Hamburg, Germany; KI681C01). Ki67 staining was visualized by goat-anti-rabbit Alexa Fluor 647 secondary antibody (1:200; Invitrogen). For detection of BrdU, mice received intraperitoneal injection of 200 µl BrdU solution (32.5 mM) (BrdU Flow Kit, BD Biosciences; Franklin Lakes, NJ, USA; 552598) 3h prior to the experiment. Intranuclear BrdU was detected with anti–BrdU-Alexa Fluor 647 (PRB-1; 1:50; Abcam) in permeabilized cells after treatment with DNase IV (Sigma-Aldrich; D5025-15KU). For all experiments fluorescence minus one and isotype controls were carried out. Macrophage and leukocyte subsets as well as BrdU^+^ and Ki67^+^ cells were gated according to isotype controls (exemplary gating strategies are provided in **Supplementary Figure 4**. M1 macrophages were defined as CD11c+CD206- whereas M2 macrophages were defined as CD11c-CD206+.

Flow cytometry was performed on an LSR II (BD Biosciences) with FACSDiva software 8.0. Gating was performed with FlowJo software 10.6 (Tree Star; Ashland, OR, USA).

### RNA isolation and quantitative real-time PCR analysis

RNA was extracted using TRI Reagent solution (Thermo Fischer Scientific; 15596018) and reverse transcribed into cDNA with RevertAid H Minus Reverse Transcriptase (Thermo Fischer Scientific; EP0451). mRNA expression of genes was measured on an Applied Biosystems StepOnePlus Real-Time PCR-Cycler (Applied Biosystems; Waltham, MA, USA) with Hot FirePol EvaGreen qPCR Mix Plus (ROX) (Biotium Iinc.; Hayward, CA, USA; 31077). Relative gene expression was adjusted to *Ipo8* and calculated according to ΔΔCt method by Pfaffl (33). Primers for relative gene expression analysis are given in **Supplementary Table 2**.

### RNA sequencing

BMDMs were treated with IL-6, IL-13 or PBS for 48h. Afterwards 500.000 – 1.000.000 cells per condition were harvested and stored at −80 °C in TRIzol (Thermo Fischer Scientific) until sequencing. RNA sequencing was performed by Single Cell Discoveries (Utrecht, The Netherlands). RNA extraction and library preparation followed the CEL-seq2 protocol with a sequencing depth of 10 million reads/sample. RNASeq data are available at SRA database, accession number PRJNA971096.

### Differential gene expression analysis

For RNA-seq data analyses low quality read ends were clipped off using Cutadapt (v 1.14) (46). Subsequently, the processed sequencing reads were aligned to the murine reference genome (UCSC mm39) using HiSat2 (v 2.1.0) (47). Samtools (v 1.10) were used to extract primary alignments and to index the resulting bam-files (48). FeatureCounts (v 2.0.0) was used for summarizing gene-mapped reads (49). ENSEMBL (GRCm39 v105) was used as annotation basis (50). Differential gene expression was determined using the R package edgeR (v 3.38.4) utilizing trimmed mean of M-values (TMM) normalization (51, 52). In order to account for biases in the expression values introduced by different batches, blocking was used to reduce these effects. A false discovery rate (FDR) value below 0.05 was considered as threshold for the determination of differential gene expression.

### Gene set enrichment and gene ontology analysis

Gene set enrichment analysis (GSEA) was performed using the R-package clusterProfiler (v 4.4.4) and MSigDB gene sets (v7.5.1) utilizing the fgsea algorithm and setting the exponent parameter to 0 for unweighted analyses of log2 fold change sorted gene lists obtained from differential gene expression analyses (53, 54). Since the used MsigDB gene sets contain human gene symbols, the human homologous symbols of the respective mouse genes were obtained via the R-package biomaRt (v 2.52.0) using ENSEMBL v105 as reference data set (55). Gene ontology (GO) analysis was performed with datasets from differential gene expression with FDR <0.05 by using g:Profiler (56).

### Statistical analysis

Statistical and analysis and data visualization was performed with Prism 9.0 software (GraphPad Software, La Jolla, CA, USA). Data in graphs and charts are given as means ± SE. Data sets were tested for statistical outliers and normal distribution before testing for statistical significance. Data sets analyzed with paired or unpaired Student´s t-tests or one-way ANOVA followed by Dunnett’s *post hoc* test. *P* values <0.05 were considered as significant.

## Data availability statement

The datasets presented in this study can be found in online repositories. The names of the repository/repositories and accession number(s) can be found in the article/**Supplementary Material**.

## Ethics statement

The animal study was approved by Landesmininsterium Sachsen TVV 11/18. The study was conducted in accordance with the local legislation and institutional requirements.

## Author contributions

MGe designed the study. JA and LA carried out the experiments with help from JF (ipITT/ipGTT), CH and MK (genotyping) and AL (RNA sequencing). JA, JB and LA analyzed the data with help from MGe, JF (ipITT/ipGTT) and MGl (RNA Sequencing). FW generated and characterized transgenic mice. JA, JB and MGe wrote the paper. All authors approved the final version of the manuscript.

## Supplementary material

Supplementary Figure 1 Comparison of female and male *Il6ra* ^fl/fl^ and *Il6ra* ^Δmyel^ mice under NCD. **(A, B)** Organ weights of (A, n=10-15) female NCD and (B, n=6-7) male NCD *Il6ra* ^fl/fl^ and *Il6ra* ^Δmyel^ mice. **(C)** Food intake in g per day of male and female *Il6ra* ^fl/fl^ and *Il6ra* ^Δmyel^ mice in NCD over 5 days (n=9). **(D-G)** Blood glucose measurements after ipITT **(D, E)** and ipGTT **(F, G)** of male NCD *Il6ra* ^fl/fl^ and *Il6ra* ^Δmyel^ mice (n=15). Data are presented as mean ± SEM. **p < 0.01, ****p < 0.0001.

Supplementary Figure 2 Leukocytes, ATM content and gene expression in whole AT adipose tissue of *Il6ra* ^fl/fl^ and *Il6ra* ^Δmyel^ mice. **(A)** Relative gene expression of selected interleukin receptors, interleukins as well as activation and proliferation markers in obese AT of male *Il6ra* ^fl/fl^ and *Il6ra* ^Δmyel^ mice (n=5-7). *Ipo8* served as internal control. **(B)** Leukocytes (CD45+) in percent of living cells from the stromal vascular fraction (SVF) of female and male NCD and male HFD *Il6ra* ^fl/fl^ and *Il6ra* ^Δmyel^ mice measured by flow cytometry (n=6-12). **(C)** Comparison of ATMs (CD45+F4/80+; percentage of living SVF cells) in female and male NCD and HFD *Il6ra* ^fl/fl^ and *Il6ra* ^Δmyel^ mice (n=6-12). Population of **(D)** CD11c-CD206- and **(E)** CD11c+CD206+ ATMs in AT of female and male NCD as well as male HFD *Il6ra* ^fl/fl^ or *Il6ra* ^Δmyel^ mice (n=.6-12). **(F)** BrdU incorporation in ATMs (CD45+F4/80+) of obese *Il6ra* ^fl/fl^ and *Il6ra* ^Δmyel^ mice given as percentage of all ATMs (n=9-10). **(G)** Representative flow cytometry plot for BrdU incorporation in ATMs (CD45+F4/80+) of obese *Il6ra* ^fl/fl^ and *Il6ra* ^Δmyel^ mice. Data are presented as mean ± SEM. **p < 0.01.

Supplementary Figure 3 IL-6 signaling does not interfere with T-cell subsets in obese AT. **(A-C)** Flow cytometry analysis of (A) CD4+CD8- T-helper cells and **(B)** CD4-CD8+ cytotoxic T-cells in percent of overall ATT (CD45+CD3+) in female and male *Il6ra* ^fl/fl^ and *Il6ra* ^Δmyel^ mice after NCD and HFD (n=6-12). **(C)** Ratio of CD4+CD8- and CD4-CD8+ ATTs in *female* and male *Il6ra* ^fl/fl^ and *Il6ra* ^Δmyel^ mice after NCD and HFD measured by flow cytometry (n=6-11). **(D)** Representative flow cytometry plots of ATTs in obese and healthy AT of female and male *Il6ra* ^fl/fl^ and *Il6ra* ^Δmyel^ mice. Data are presented as mean ± SEM.

Supplementary Figure 4 Gating strategy for flow cytometry. **(A)** Gating strategy for flow cytometric analysis of ATMs. Empty channel represents an unstained channel for eliminating autofluorescence. 7-AAD was used to identify permeabilized single cells. **(B)** Gating strategy for flow cytometry analysis of ATTs.

Supplementary Figure 5 Heat maps of RNA sequencing data. BMDMs were isolated from *Il4ra* ^-/-^ mice and wildtype (*Il4ra* ^+/+^) mice and stimulated with IL−13 or IL-6 (20 ng/mL) for 48h (n=4). Differential gene expressions were determined from RNA bulk sequencing data. Datasets of the 100 most differentially expressed genes (FDR < 0.05) were compared between genotypes and conditions and displayed in heat maps sorted by FDR from low to high.

Supplementary Table 1 Detailed numbers for comparison of *Il6ra* ^fl/fl^ and *Il6ra* ^Δmyel^ mice. Detailed numbers for comparison of female and male *Il6ra* ^fl/fl^ and *Il6ra* ^Δmyel^ mice under NCD or HFD concerning body and organ weights, ATM and ATT populations and measurements of free fatty acids (FFA), Cholesterol (HDL, LDL) and Triglycerides as well as histological features.

Supplementary Table 2 List of primers used for qRT-PCR. for (forward), rev (reverse).

Supplementary Table 3 Results of Gene Ontology (GO) term enrichment analysis. Significantly up- or down regulated GO terms for respective comparisons are given with GO class, term name, term ID, adjusted p-value and GO size.

Supplementary Table 4 Results of gene set enrichment analysis (GSEA). Significantly up- or down regulated gene sets for respective comparisons are given with gene set name, size, enrichment score, NES, p-value and adjusted p-value.
