# Supplementary material for: IL-6 signaling drives self-renewal and alternative activation of adipose tissue macrophages
Source: Front Immunol. 2024 Feb 28;15:1201439. doi: 10.3389/fimmu.2024.1201439 (PMC10933059; doi:10.3389/fimmu.2024.1201439)
Supplement: Supplementary Figure 1 — Comparison of female and male Il6ra fl/fl and Il6ra Δmyel mice under NCD. (A, B) Organ weights of (A, n=10-15) female NCD and (B, n=6-7) male NCD Il6ra fl/fl and Il6ra Δmyel mice. (C) Food intake in g per day of male and female Il6ra fl/fl and Il6ra Δmyel mice in NCD over 5 days (n=9). (D-G) Blood glucose measurements after ipITT (D, E) and ipGTT (F, G) of male NCD Il6ra fl/fl and Il6ra Δmyel mice (n=15). Data are presented as mean ± SEM. **p < 0.01, ****p < 0.0001. [file DataSheet_1.pdf]

SUPPLEMENTARY MATERIAL

IL-6 signaling as a driver of self-renewal and alternative activation of adipose tissue macrophages

Jan Ackermann[1,2,†], Lilli Arndt[1,2,†], Janine Fröba[1], Andreas Lindhorst[1], Markus Glaß[3], Michaela Kirstein[2], Constance Hobusch[1], F. Thomas Wunderlich[4], Julia Braune[1,†], Martin Gericke[1,2,†*]

[1] Institute of Anatomy, Leipzig University, Leipzig, Germany,
[2] Institute of Anatomy and Cell Biology, Martin-Luther-University Halle-Wittenberg, Halle (Saale), Germany,
[3] Institute of Molecular Medicine, Martin Luther University Halle-Wittenberg, Charles Tanford protein center, Halle (Saale), Germany
[4] Max-Planck-Institute for Metabolism Research, Research Group for Obesity and Cancer, Cologne, Germany

[†] These two authors contributed equally to this work.

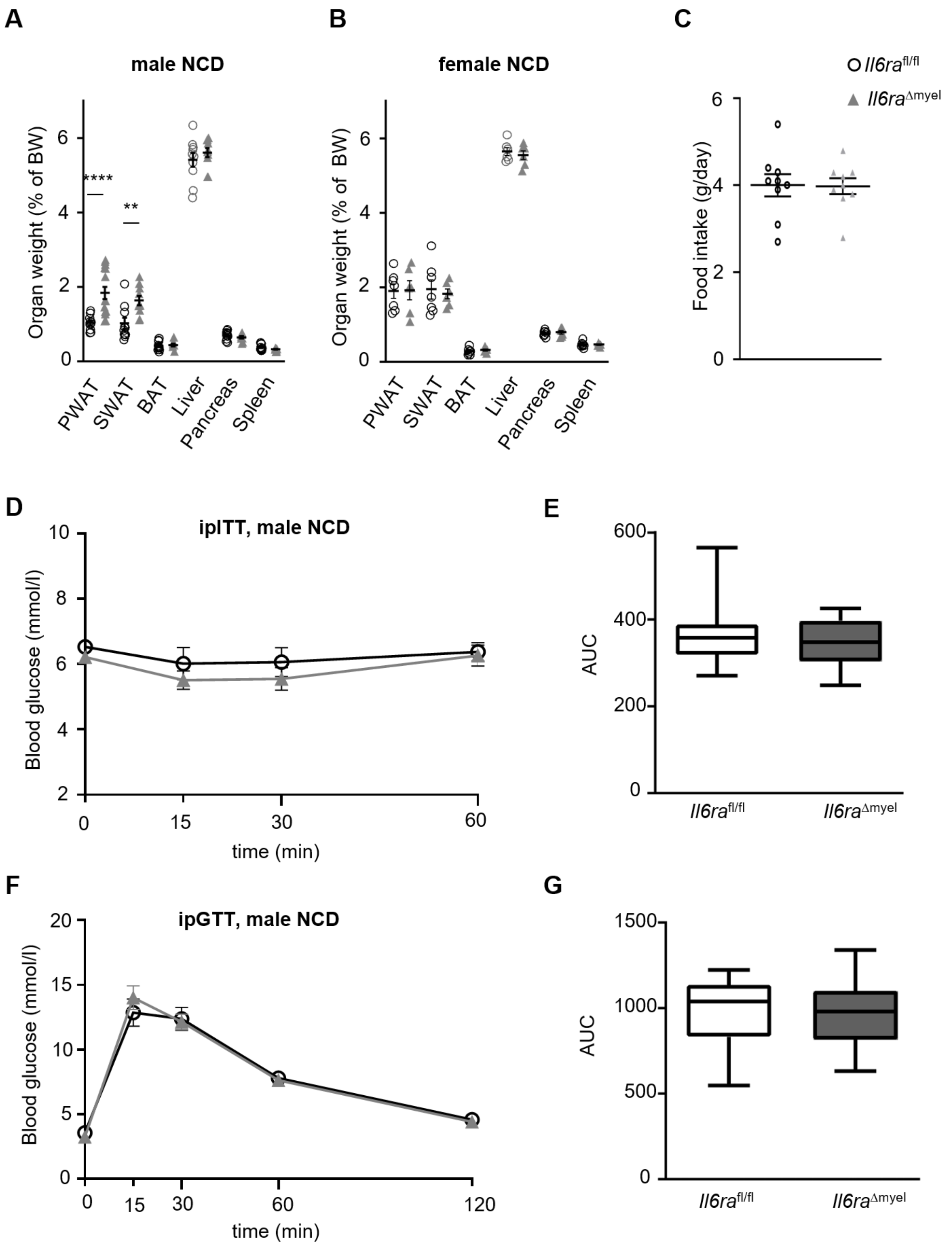

SUPPLEMENTARY FIGURE 1

Comparison of female and male *Il6ra*$^{fl/fl}$ and *Il6ra*$^{\Delta myel}$ mice under NCD. (**A, B**) Organ weights of (**A**, n=10-15) female NCD and (**B**, n=6-7) male NCD *Il6ra*$^{fl/fl}$ and *Il6ra*$^{\Delta myel}$ mice. (**C**) Food intake in g per day of male and female *Il6ra*$^{fl/fl}$ and *Il6ra*$^{\Delta myel}$ mice in NCD over 5 days (n=9). (**D-G**) Blood glucose measurements after ipITT (**D, E**) and ipGTT (**F, G**) of male NCD *Il6ra*$^{fl/fl}$ and *Il6ra*$^{\Delta myel}$ mice (n=15). Data are presented as mean ± SEM. $^{**}p < 0.01$, $^{****}p < 0.0001$.

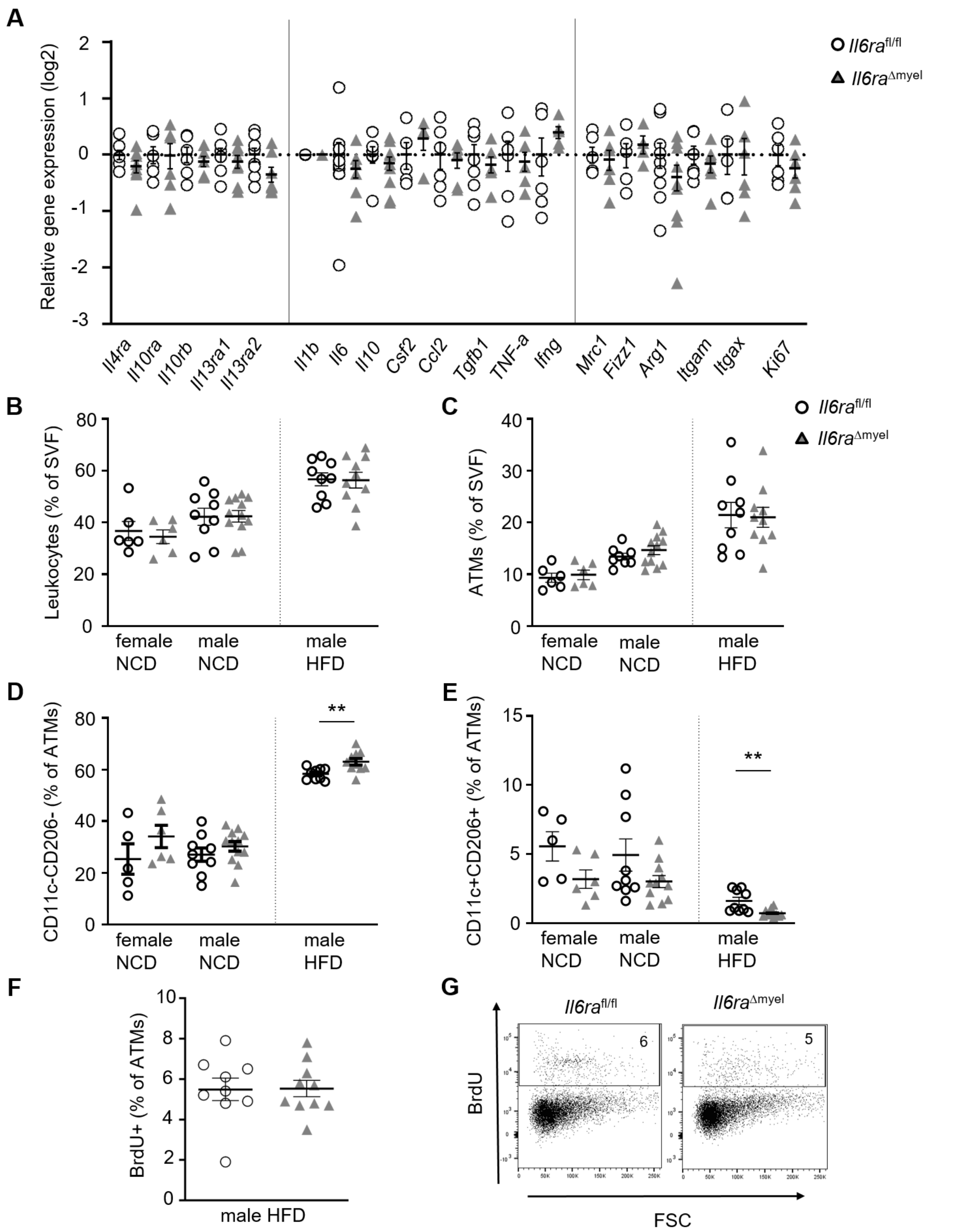

SUPPLEMENTARY FIGURE 2

Leukocytes, ATM content and gene expression in whole AT of *Il6ra*$^{fl/fl}$ and *Il6ra*^{Δmyel} mice. (**A**) Relative gene expression of selected interleukin receptors, interleukins as well as activation and proliferation markers in obese AT of male *Il6ra*$^{fl/fl}$ and *Il6ra*^{Δmyel} mice (n=5-7). *Ipo8* served as internal control. (**B**) Leukocytes (CD45+) in percent of living cells from the stromal vascular fraction (SVF) of female and male NCD and male HFD *Il6ra*$^{fl/fl}$ and *Il6ra*^{Δmyel} mice measured by flow cytometry (n=6-12). (**C**) Comparison of ATMs (CD45+F4/80+; percentage of living SVF cells) in female and male NCD and

HFD *Il6ra*$^{fl/fl}$ and *Il6ra*$^{\Delta myel}$ mice (n=6-12). Population of (**D**) CD11c-CD206- and (E) CD11c+CD206+ ATMs in AT of female and male NCD as well as male HFD *Il6ra*$^{fl/fl}$ or *Il6ra*$^{\Delta myel}$ mice (n=.6- 12). (**F**) BrdU incorporation in ATMs (CD45+F4/80+) of obese *Il6ra*$^{fl/fl}$ and *Il6ra*$^{\Delta myel}$ mice given as percentage of all ATMs (n=9-10). (**G**) Representative flow cytometry plot for BrdU incorporation in ATMs (CD45+F4/80+) of obese *Il6ra*$^{fl/fl}$ and *Il6ra*$^{\Delta myel}$ mice. Data are presented as mean ± SEM. **p < 0.01.

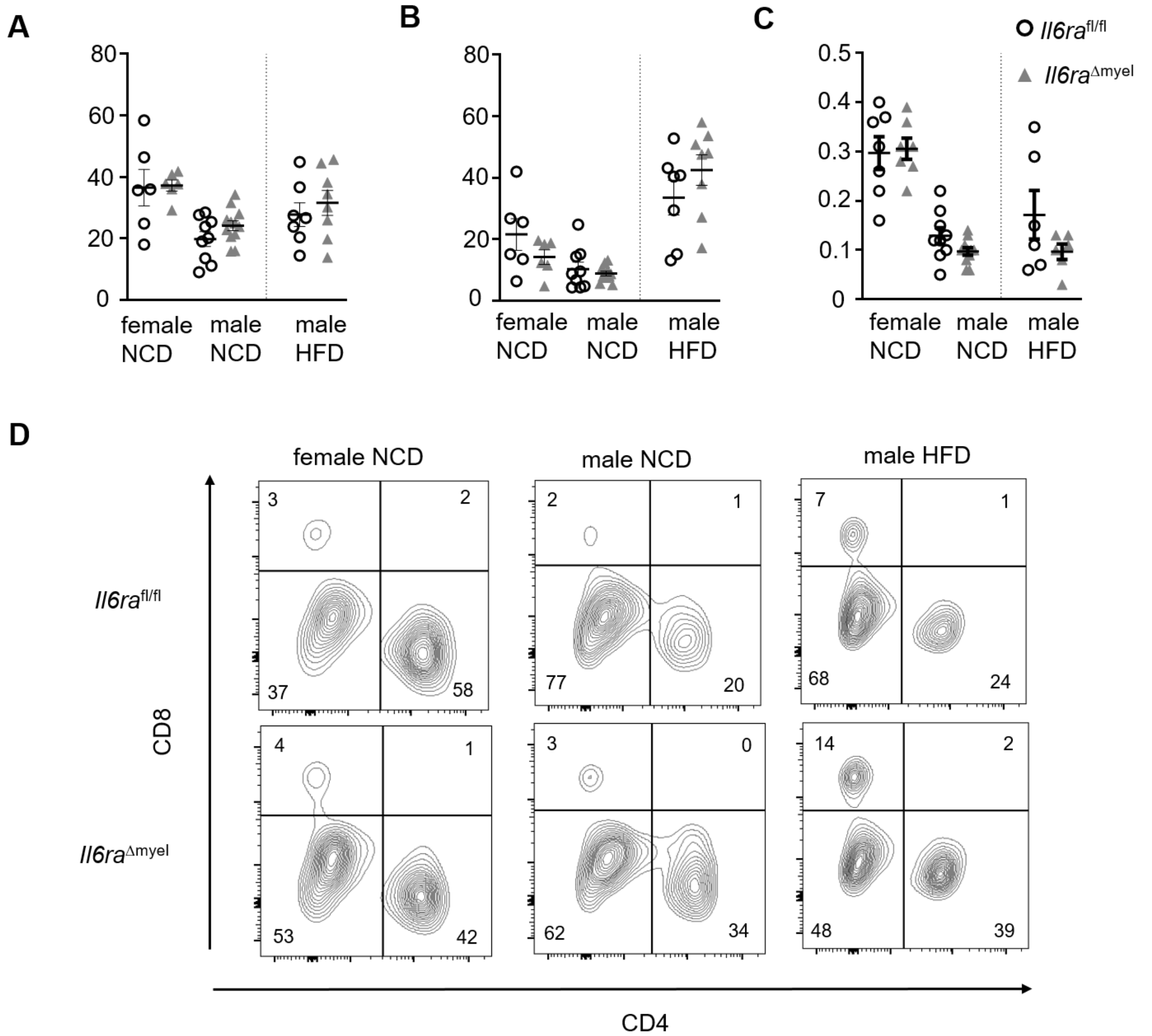

SUPPLEMENTARY FIGURE 3

IL-6 signaling does not interfere with T-cell subsets in obese AT. (**A-C**) Flow cytometry analysis of (**A**) CD4+CD8- T-helper cells and (**B**) CD4-CD8+ cytotoxic T-cells in percent of overall ATT (CD45+CD3+) in female and male *Il6ra*$^{fl/fl}$ and *Il6ra*$^{\Delta myel}$ mice after NCD and HFD (n=6-12). (**C**) Ratio of CD4+CD8- and CD4-CD8+ ATTs in female and male *Il6ra*$^{fl/fl}$ and *Il6ra*$^{\Delta myel}$ mice after NCD and HFD measured by flow cytometry (n=6-11). (**D**) Representative flow cytometry plots of ATTs in obese and healthy AT of female and male *Il6ra*$^{fl/fl}$ and *Il6ra*$^{\Delta myel}$ mice. Data are presented as mean ± SEM.

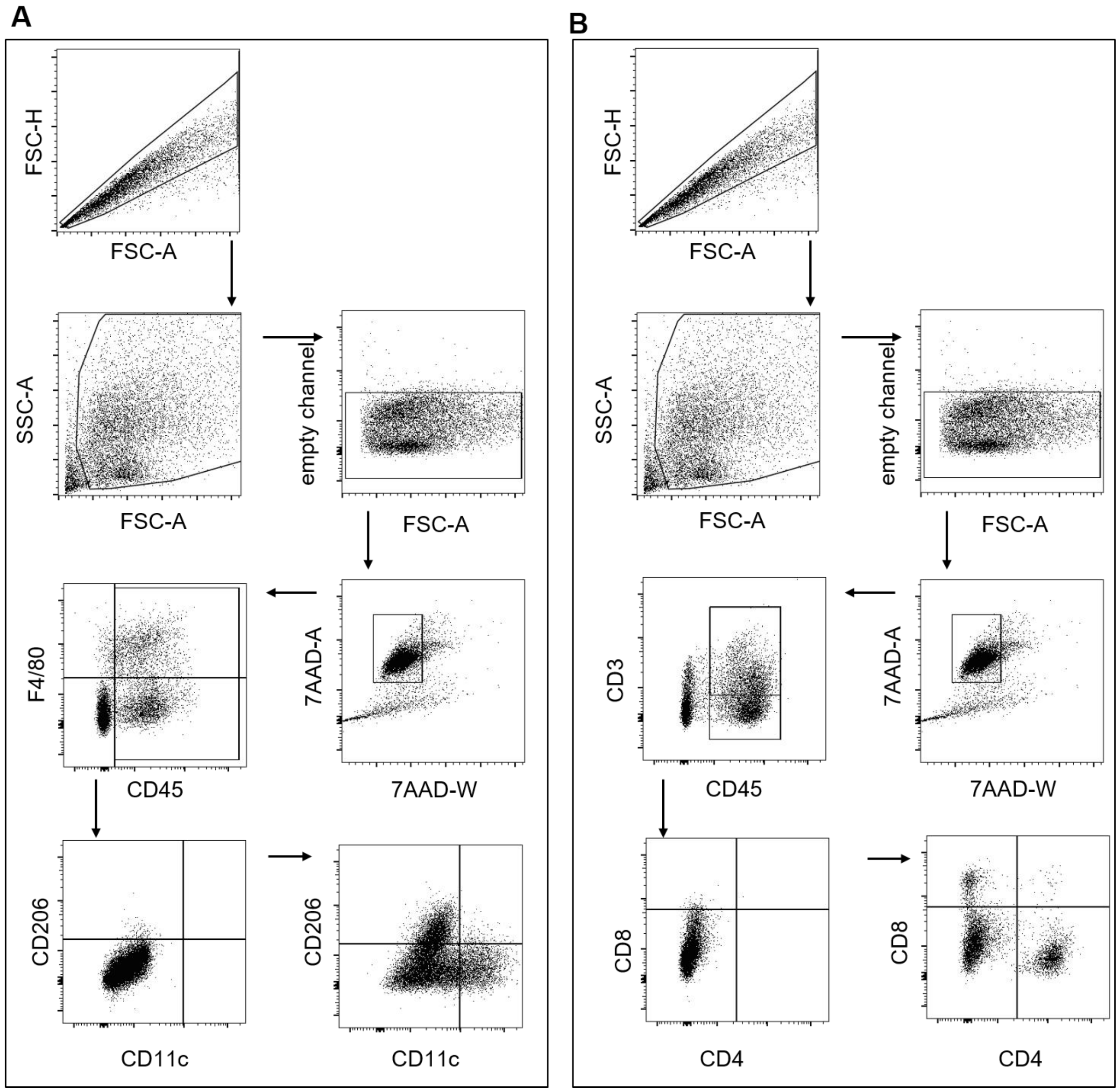

SUPPLEMENTARY FIGURE 4

Gating strategy for flow cytometry. (**A**) Gating strategy for flow cytometric analysis of ATMs. Empty channel represents an unstained channel for eliminating autofluorescence. 7-AAD was used to identify permeabilized single cells. (**B**) Gating strategy for flow cytometry analysis of ATTs**.**

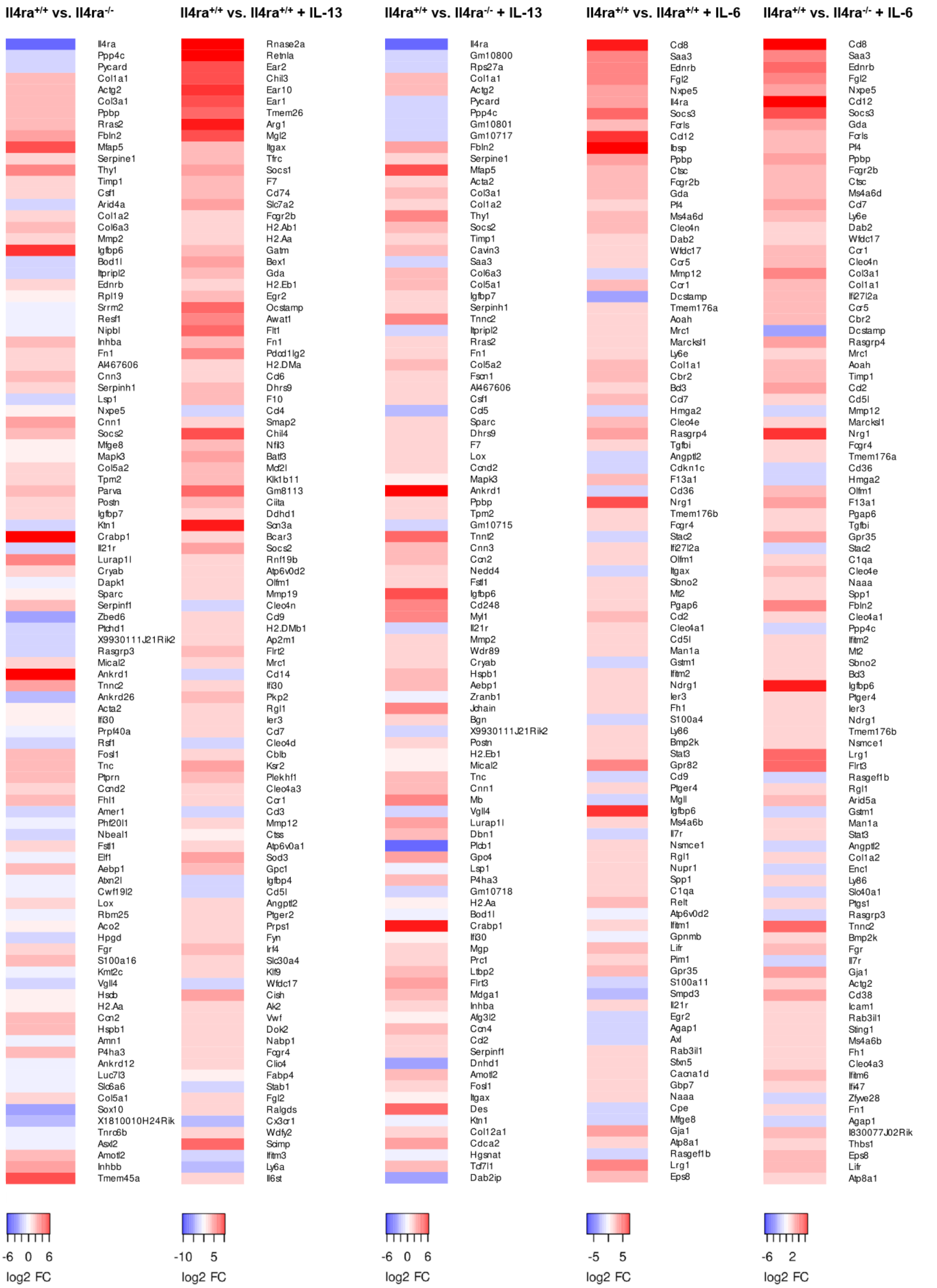

SUPPLEMENTARY FIGURE 5

Heat maps of RNA sequencing data. BMDMs were isolated from *Il4ra*$^{-/-}$ mice and wildtype (*Il4ra*$^{+/+}$) mice and stimulated with IL−13 or IL-6 (20 ng/mL) for 48h (n=4). Differential gene expressions were determined from RNA bulk sequencing data. Datasets of the 100 most differentially expressed genes (FDR < 0.05) were compared between genotypes and conditions and displayed in heat maps sorted by FDR from low to high.
